# Monoclonal antibody‐mediated immunosuppression enables long‐term survival of transplanted human neural stem cells in mouse brain

**DOI:** 10.1002/ctm2.1046

**Published:** 2022-09-13

**Authors:** Lisa M. McGinley, Kevin S. Chen, Shayna N. Mason, Diana M. Rigan, Jacquelin F. Kwentus, John M. Hayes, Emily D. Glass, Evan L. Reynolds, Geoffrey G. Murphy, Eva L. Feldman

**Affiliations:** ^1^ Department of Neurology University of Michigan Ann Arbor Michigan USA; ^2^ Department of Neurosurgery University of Michigan Ann Arbor Michigan USA; ^3^ Department of Molecular and Integrative Physiology University of Michigan Ann Arbor Michigan USA; ^4^ Michigan Neuroscience Institute University of Michigan Ann Arbor Michigan USA

**Keywords:** Alzheimer's disease, antibodies, cell tracking, immunosuppression therapy, monoclonal, stem cell transplantation

## Abstract

**Background:**

As the field of stem cell therapy advances, it is important to develop reliable methods to overcome host immune responses in animal models. This ensures survival of transplanted human stem cell grafts and enables predictive efficacy testing. Immunosuppressive drugs derived from clinical protocols are frequently used but are often inconsistent and associated with toxic side effects. Here, using a molecular imaging approach, we show that immunosuppression targeting costimulatory molecules CD4 and CD40L enables robust survival of human xenografts in mouse brain, as compared to conventional tacrolimus and mycophenolate mofetil.

**Methods:**

Human neural stem cells were modified to express green fluorescent protein and firefly luciferase. Cells were implanted in the fimbria fornix of the hippocampus and viability assessed by non‐invasive bioluminescent imaging. Cell survival was assessed using traditional pharmacologic immunosuppression as compared to monoclonal antibodies directed against CD4 and CD40L. This paradigm was also implemented in a transgenic Alzheimer's disease mouse model.

**Results:**

Graft rejection occurs within 7 days in non‐immunosuppressed mice and within 14 days in mice on a traditional regimen. The addition of dual monoclonal antibody immunosuppression extends graft survival past 7 weeks (*p* < .001) on initial studies. We confirm dual monoclonal antibody treatment is superior to either antibody alone (*p* < .001). Finally, we demonstrate robust xenograft survival at multiple cell doses up to 6 months in both C57BL/6J mice and a transgenic Alzheimer's disease model (*p* < .001). The dual monoclonal antibody protocol demonstrated no significant adverse effects, as determined by complete blood counts and toxicity screen.

**Conclusions:**

This study demonstrates an effective immunosuppression protocol for preclinical testing of stem cell therapies. A transition towards antibody‐based strategies may be advantageous by enabling stem cell survival in preclinical studies that could inform future clinical trials.

## Graphical abstract

– Traditional immunosuppression with tacrolimus and mycophenolate mofetil did not sustain human neural stem cell xenograft survival in mouse brain for more than 2 weeks.
– Immunosuppression with monoclonal antibodies targeting CD4 and CD40L enabled long‐term persistence of human stem cell transplants.
– Transplants survive past 28 weeks in C57BL/6 mice and the 5XFAD animal model of Alzheimer's disease.

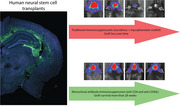

## 1 BACKGROUND

Transplantation of human stem cells in the central nervous system (CNS) represents a rapidly developing, multifaceted approach for the treatment of neurological disorders.^1^, ^2^ Preclinical studies show that stem cell transplantation can improve disease‐related pathology and neurologic function, and advances have led to clinical testing in patients with stroke, Parkinson's disease, amyotrophic lateral sclerosis and Alzheimer's disease.^3^, ^4^, ^5^, ^6^ Established human neural stem cell (hNSC) lines are increasingly utilized in clinical trials^7^, ^8^, ^9^, ^10^, ^11^in lieu of autologous sources of stem cells (e.g., mesenchymal stem cells). There are many benefits to using a hNSC line, including commitment to neuronal/glial fates, the ability to characterize and/or modify cells ex vivo, and amenability to large‐scale production, thus reducing cost and increasing accessibility for patients. However, US Food and Drug Administration approval for biologic therapies requires preclinical efficacy testing in small animals, necessitating xenogeneic transplant paradigms. Given this requirement, host rejection of transplanted cells and the reliance on immunosuppressive agents to prevent an immune response, even in human allogenic transplant paradigms, remain significant challenges.^12^, ^13^, ^14^, ^15^

Traditionally considered an ‘immunoprivileged’ site, it is now known that CNS antigens are transported to peripheral lymph nodes, and activated T cells can cross the blood brain barrier.^16^, ^17^ In the context of xenogeneic cell transplantation in the CNS, recipient CD4^+^ T cells appear to play a major role, capable of recognizing donor antigens, triggering the adaptive immune response and consequent graft rejection.^18^, ^19^, ^20^ Costimulatory signalling, for example via CD40‐CD40L interactions, appears to be a necessary component of xenograft rejection.^21^, ^22^ This represents a substantial hurdle to ensuring robust survival of human cells in xenograft experimental models.^21^, ^23^ Immunosuppressive drug regimens derived from clinical protocols for solid organ transplant have been deployed to prevent xenograft rejection and include steroids and inhibitors of calcineurin, inosine monophosphate dehydrogenase, interleukins and tumour necrosis factor α.^24^, ^25^ In terms of xenogeneic grafts, the long‐term effectiveness of these immunosuppressive drugs in preventing cytotoxic immune‐mediated rejection of human stem cell transplants in the CNS is not well established. Furthermore, standard non‐specific immunosuppression paradigms often do not sufficiently overcome host immune responses, with transient survival of transplanted human cells and subsequent graft rejection within 4–6 weeks in rodents.^26^, ^27^, ^28^, ^29^, ^30^, ^31^, ^32^ More effective and specific immunosuppressive regimens are needed for robust xenograft survival in rodent models to extend experimental timelines, enable evaluation of maximum therapeutic effects and mitigate effects of stem cell loss.

Emerging approaches include the use of monoclonal antibodies (mAbs) targeted to specific immune cell populations and costimulatory pathways involved in the immune response.^33^, ^34^, ^35^, ^36^, ^37^ In preclinical studies, various combinations of mAbs have been used to promote immune tolerance in transplantation experiments. Although this approach seems promising, the application of long‐term mAb‐based immunosuppressive regimens to CNS stem cell transplantation therapy is unknown. This prompted us to investigate a mAb‐based immunosuppressive regimen consisting of anti‐CD4 and anti‐CD40L mAb co‐stimulatory blockade in the context of preclinical intracranial hNSC transplantation. Using real‐time in vivo bioluminescent imaging (BLI), we demonstrate that this mAb‐based immunosuppressive regimen enables robust and durable survival and extensive migration of a clinically relevant hNSC line following intracranial transplantation in both normal and Alzheimer's disease mouse models.

## 2 METHODS

### 2.1 In vitro generation of hNSC‐luc^±^/green fluorescent protein^±^ cells

hNSC lines (HK532‐CAG‐IGF1, previously assessed for intracranial transplantation)^38^, ^39^ were supplied by Seneca BioPharma, Inc. (Germantown, USA) and cultured as previously described.^40^, ^41^ Stable reporter gene expression was achieved in hNSCs with a lentivirus vector (LV‐Luc2‐P2A‐EmGFP, # LV050‐L, Imanis Life Sciences, Rochester, USA) encoding firefly luciferase (luc) and emerald green fluorescent protein (GFP). Briefly, lentivirus was added to hNSCs at approximately 70% confluence in growth media and incubated for approximately 16 h in normal culture conditions (5% O_2_, 5% CO_2_, 37°C). After incubation, cells were washed three times in growth media to remove virus. Transduction efficiency was monitored under fluorescence microscopy for GFP expression. Luciferase expression was measured at 48 h post‐transduction using a luciferase activity assay per manufacturer protocols (Promega, Madison, USA). For transplantation, a bank of hNSC‐luc^+^/GFP^+^ transduced at multiplicity of infection (MOI) 25 was expanded and stored in liquid nitrogen.

### 2.2 Stem cell transplantation

We assessed viability of hNSC transplants in C57BL/6J mice (Jackson Laboratory, Bar Harbor, USA) as well as the 5XFAD transgenic mouse (Jackson Laboratory), a commonly used model of Alzheimer's disease.^42^, ^43^, ^44^These mice harbour two humanized transgenes: (1) the amyloid‐β precursor protein gene with the Swedish, Florida and London mutations and (2) the presenilin‐1 (*PSEN1*) gene harbouring the M146L and L286V mutations. It is important to note here that we^45^ and others^46^ have demonstrated that the neurological and behavioural phenotypes on the C57BL/6J genetic background are less severe and occur at later timepoints when compared to that observed on the original C57BL6/SJLF1 hybrid background.^42^ Mice were randomly assigned to treatment groups for each experiment. Sample sizes are specified in figures and/or figure legends, and outliers are included. Personnel performing BLI, immunohistochemistry, microscopy, complete blood counts (CBCs), flow cytometry, ELISA and toxicity screen analysis were blinded to the treatment groups. All animal procedures were approved by the University of Michigan Institutional Animal Care and Use Committee (PRO00010247) and performed according to University of Michigan guidelines and state and federal regulations, including the NIH Guide for the Care and Use of Laboratory Animals.

Intracranial transplantation was performed on 8‐10‐week‐old male C57BL/6J or 5XFAD mice (Jackson Laboratory) using our established stereotactic approach.^39^, ^40^, ^41^, ^47^ Briefly, mice were anesthetized with 2% isoflurane and placed in a standard Kopf stereotactic frame (David Kopf Instruments, Tujunga, USA). hNSC‐luc^+^/GFP^+^ vials were thawed and cultured in N2b Growth Media (Seneca Biopharma) supplemented with basic fibroblast growth factor. At the same passage number, cells were harvested with 0.25% trypsin followed by addition of soybean trypsin inhibitor (Invitrogen, Waltham, USA, 0.5 mg/ml). hNSCs were pelleted by centrifugation, then resuspended in hibernation media (Seneca BioPharma) and trypan blue exclusion ensured transplantation of >90% viable cells. hNSCs were delivered by bilateral injection to the fimbria fornix of the hippocampus at three sites per hemisphere (2 μl hNSC‐luc^+^/GFP^+^, total six injections) delivered to the following coordinates (bregma/lateral/ventral): −0.82/0.75/2.5, −1.46/2.3/2.9, −1.94/2.8/2.9 mm. Cell concentrations varied depending on the experiment and intended cell dose (see figure legends). Final cell doses ranged between 1.8 × 10^5^ and 9.6 × 10^5^ total cells per animal. Each injection was administered over 120 s followed by a 120 s delay prior to needle withdrawal. Cell viability was reassessed by trypan blue exclusion post‐transplantation to ensure adequate cell survival throughout the procedure.

### 2.3 Immunosuppression treatments

Body weights were collected weekly for all mice for accurate dosing. For the initial comparison of tacrolimus with mycophenolate mofetil (Tac/MMF) versus mAbs in C57BL/6J, mice were either non‐immunosuppressed or received one of several immunosuppression regimens. For Tac/MMF treatment, beginning 1 week preoperatively, mice received intraperitoneal MMF (30 mg/kg daily, Genentech USA Inc., South San Francisco, USA) until post‐operative day (POD) 7 and intraperitoneal Tac (5 mg/kg daily, Astellas Pharmas US Inc., Northbrook, USA) beginning 1 week preoperatively and continuing daily for the study duration. For animals in this experiment comparing Tac/MMF to mAbs, induction of immunosuppression by mAb therapy (20 mg/kg each, intraperitoneal) was begun on the day of surgery and given for three daily consecutive doses. Subsequent maintenance mAb treatment was given every 7 days thereafter for the study duration. Depleting mAb treatment targeted CD4 (Clone GK1.5, Rat IgG2b,κ, 20 mg/kg, Bio X Cell, Lebanon, USA) and CD40L (Clone MR‐1, Armenian Hamster IgG, 20 mg/kg, Bio X Cell). The Tac/MMF/mAb groups received the above regimens in combination.

In subsequent experiments, mAb immunosuppression began on the day prior to surgery and induction dosing continued for four daily consecutive doses followed by maintance therapy every 7 days for the study duration. This mAb regimen was administered to C57BL/6J and 5XFAD mice for hNSC dosing and long‐term tracking experiments. For comparison of both mAbs versus single mAb treatment, mice received the same mAb treatment as above, compared with anti‐CD4 mAb alone (20 mg/kg), or anti‐CD40L mAb alone (20 mg/kg) on the same dosing schedule.

### 2.4 BLI

For in vitro imaging, hNSC‐luc^+^/GFP^+^ were plated in a black‐walled, clear bottom 96‐well plate at various cell concentrations 1–2 days before imaging. Control groups included unlabelled hNSC (prepared identically to hNSC‐luc^+^/GFP^+^) and dead hNSC‐luc^+^/GFP^+^ (cells subjected to heat shock at 70°C for 3 min, verified by trypan blue staining). On the day of imaging when cells were approximately 70%–80% confluent, D‐Luciferin was added at 150 μg/ml 2 min prior to imaging. Imaging was performed using the bioluminescence protocol with open emission, an exposure time of 30 s, medium binning and 1.5 cm subject height.

In vivo BLI was performed in the Center for Molecular Imaging at the University of Michigan using the IVIS Spectrum in vivo Imaging System (PerkinElmer, Waltham, USA). The IVIS Spectrum was initialized at the start of each imaging session to cool the charge‐coupled device camera to −90°C. Mice received a single 100 μl D‐Luciferin intraperitoneal injection (resuspended at 40 mg/ml in 1× PBS) 10 min prior to imaging. Anesthesia was induced with inhaled isoflurane (2% in 100% oxygen) 6 min prior to imaging and maintained at 1.5% isoflurane for the duration of the procedure. At 2 min prior to imaging, mice were placed in prone position on the heated imaging platform inside the IVIS Spectrum chamber with integrated gas anesthesia provided through a nose cone. The following IVIS acquisition settings were used throughout the study: Exposure time 180 s; F/Stop 1; Medium Pixel Binning; Field of View C; Subject height 1.50 cm. Both Luminescent and Photograph Imaging modes were selected to render a quantitative bioluminescent signal expressed in photons/second overlaid on a photographic image of the animal under white light. BLI analysis was performed using the Living Image Software (IVIS Imaging Systems). IVIS Spectrum‐generated images were analysed using automatically generated contour regions of interest with a 10% threshold to eliminate background noise. Total flux (photons/second) data were collected and aggregated throughout the study.

### 2.5 Tissue and blood collection, histology, flow cytometry, ELISA and CBCs

Mice were euthanized by intraperitoneal pentobarbital overdose (FatalPlus, Vortech Pharmaceuticals, Dearborn, USA), and whole blood was collected from the inferior vena cava using a 23‐gauge needle. For CBCs, 100 μl of blood was placed into an EDTA‐coated 100‐μl microvette tube, gently rolled to mix and maintained at room temperature. Automated CBC analysis (element HT5, Heska, Loveland, USA) was performed at the In Vivo Animal Core at the University of Michigan within 4 h of blood draw. Following blood collection, mice were perfused with saline followed by 4% paraformaldehyde. Brains were removed and post‐fixed in 4% paraformaldehyde, cryoprotected in 30% sucrose and cryosectioned (coronal, 40 μm sections). Transplanted hNSCs were visualized in brain sections by using the 488 nm filter for GFP for hNSC‐luc^+^/GFP^+^ or by immunostaining with a primary antibody for human nuclei (HuNu; MAB1281, Millipore, Burlington, USA) for unlabelled hNSCs. Additional characterization of hNSC grafts used primary antibodies towards Nestin (ABD69, Millipore), glial fibrillary acidic protein (GFAP, Z0334, Dako, Glostrup, Denmark), Mouse IgG (4410, Cell Signaling, Danvers, USA), Mouse IgM (13‐5790‐82, Invitrogen), Mouse IgA (NB7506, Novus, Weldon Spring, USA), CD4(STJ114879‐50, St. John's Laboratory, London, UK), CD40L (STJ114971‐50, St. John's) and Iba1(091‐19741, Wako, Richmond, USA) as previously described.^39^ All fluorescent sections were counterstained with Hoechst (Pierce, Walthmam, USA).

To assess systemic toxicity associated with dual mAb treatment and/or hNSC transplantation, samples of brain, heart, liver, kidney, and pancreas of hNSC‐treated animals were submitted to the University of Michigan Unit for Laboratory Animal Medicine In Vivo Animal Core. Hematoxylin/eosin‐stained sections of formalin‐fixed paraffin‐embedded tissue from each organ were reviewed by a board‐certified veterinary pathologist for evience of organ toxicity or damage, blinded to experimental groups.

A CD40L enzyme linked immunosorbent assay (ELISA) kit (Abcam, Waltham, USA) was utilized to quantify CD40L levels in serum of mAb treated animals. Briefly, following the manufacturer's protocol, samples were diluted 1:2 and placed into a 96‐well plate pre‐coated with anti‐CD40L antibodies. Sandwich ELISA was performed by then incubating with biotin‐conjugated anti‐CD40L antibody, followed by Streptavidin‐horseradish peroxidase and tetramethylbenzidine substrate. The reaction was then stopped by Stop solution, and absorbance in each well was read on a spectrophotometer at 450‐nm wavelength (Synergy HTX, Agilent, Santa Clara, USA). Levels were compared to absorbances from a serially diluted standard of mouse CD40L provided in the kit.

To assess efficacy and off‐target effects of mAb treatment, serum samples were taken 8 days after the above four dose induction regimen of anti‐CD4 alone, anti‐CD40L alone or both mAbs, then processed for flow‐cytometry (without hNSC transplants). Briefly, red cells were lysed in blood samples, and peripheral immune cells were isolated by centrifugation. Cells were resuspended in flow cytometry buffer and Fc receptors blocked (TruStain FcX blocking solution, BioLegend, San Diego, USA). Cells were then stained with APC‐CD45, FITC‐CD3, BV421‐CD8 and APC‐Cy7 CD4 antibody (BioLegend) at 1:100 dilution, then fixed with BD Stabilizing Fixative (BD Biosciences, Franklin Lakes, NJ). Flow cytometry was performed on a BD LSRFortessa flow cytometer with FACSDiva software (BD Biosciences) and analysed with FlowJo software (FloJo LLC, Ashland, USA). Lymphocytes were initially gated by low side scatter properties then gated for CD4^+^ T cells, and the number of SSC^low^CD4^+^ cells were counted as a percentage of total lymphocytes as has been reported previously.^48^, ^49^, ^50^, ^51^, ^52^

### 2.6 Morris water maze

The Morris water maze is a well‐established method of assessing spatial memory in animal models. Briefly, at 11 months of age, animals are placed in a pool of opaque water with visual cues placed around the perimeter of the pool. Groups included wild‐type mice (WT) with no treatment as well as 5XFAD mice with no treatment, 5XFAD mice that received biweekly injections of saline or CD4/CD40L mAb. In groups receiving saline or mAb injections, intraperitoneal injections began 4 weeks prior to initiation of behavioural testing. A submerged hidden platform is placed in one quadrant, which provides an escape from water. The location of the hidden platform can be deduced by spatial relationship to the surrounding visual cues, and latency for animals to swim towards and find the hidden platform reduces over repeated trials as animals learn the spatial relationship between the platform and visual cues (four trials per day for 12 days). A positive control with visible platform is performed on the 14th day. Subsequently, to test long‐term reference memory, probe trials (with the platform removed) were conducted prior to start of training on day 4 and 24 h after the end of training (day 13). Time spent probing each quadrant of the pool is measured: mice that are successful in spatial learning and memory will spend a disproportionate amount of time searching for the platform in the quadrant where it was previously placed, whereas impaired spatial memory results in only 25% of time spent in the correct quadrant as a matter of chance.

### 2.7 Statistical analysis

All statistical analyses were performed using GraphPad Prism 8 (GraphPad Software Inc., La Jolla, USA), or R. Statistical significance was determined using an alpha‐level of 0.05. Brown‐Forsythe F‐tests were used to compare variances and determine distribution. Data were analysed by parametric *t*‐test, one‐way analysis of variance (ANOVA) with Tukey's post‐test for comparisons of multiple groups, or Pearson's correlation. Analysis of repeated BLI measurements was performed using a linear mixed effects model with random mouse‐specific intercepts to determine the association between changes in total flux during follow‐up as a function of treatment group. Specifically, the mixed models included a treatment effect, a linear follow‐up time effect and a time by treatment group interaction effect. The mixed effects models were fit using the lmerTest package in R software version 3.5.2, and model parameter estimates were determined using the maximum likelihood method.^53^ *T*‐tests calculated using Satterthwaite's degrees of freedom method were evaluated to assess differences in total flux between treatment groups during follow‐up. Since total flux was heavily skewed, outcomes were log transformed as log(Flux+1). We performed an available‐case analysis and included all information in the mixed effects models, even those without complete data. Comparison between mouse strains was performed using Wilcoxon‐Mann‐Whitney Tests. Exact *n* values, *p* values and test specifics for each experiment are included in the figure legends.

## 3 RESULTS

### 3.1 Using BLI to assess transplanted cell survival

**FIGURE 1:**
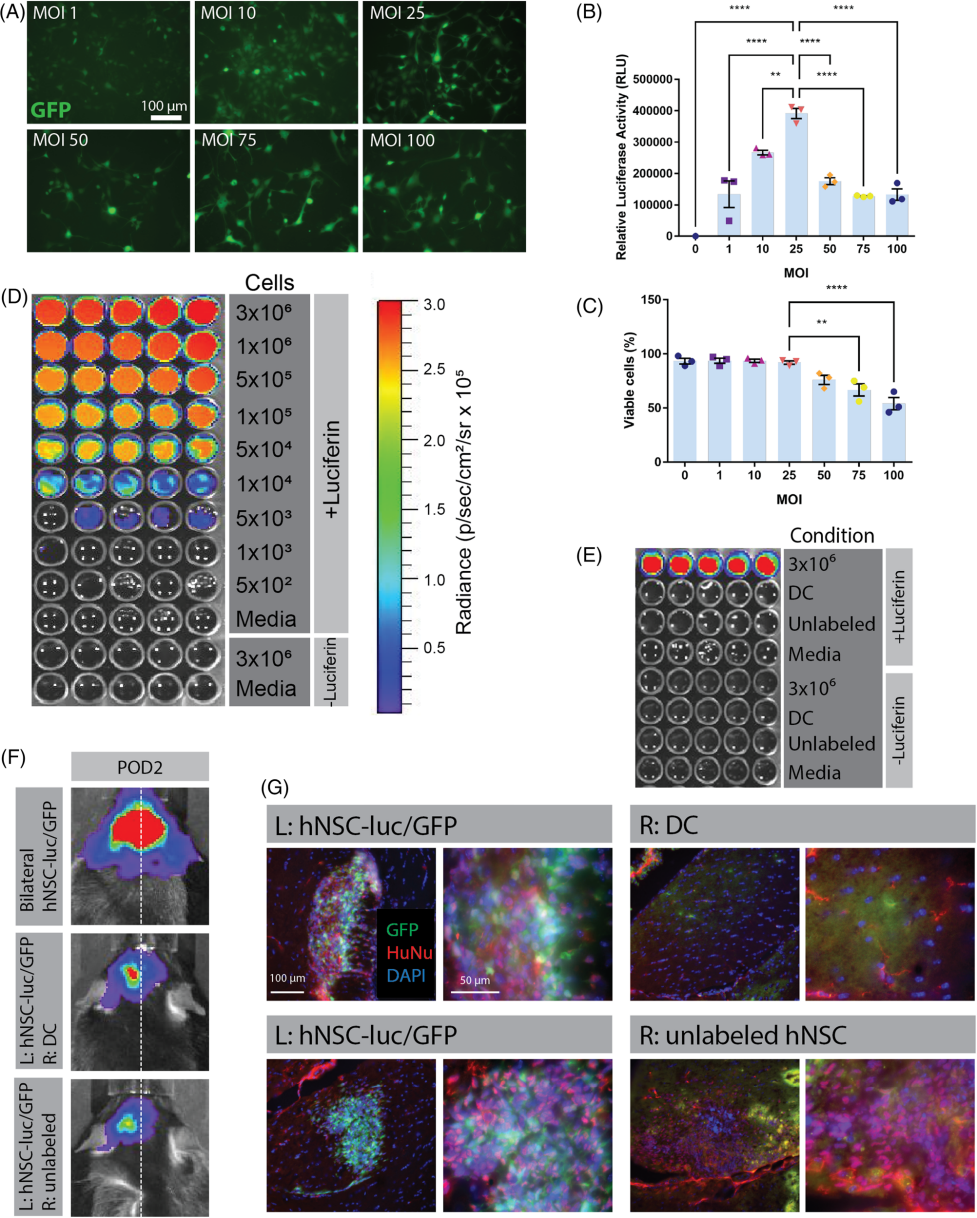
Development and validation of bioluminescent imaging (BLI) to assess transplanted human neural stem cell (hNSC) graft viability in vivo. (A) Fluorescence microscopy of green fluorescent protein (GFP) expression in hNSCs modified to express a dual reporter luc^+^/GFP^+^ vector at increasing multiplicity of infection (MOI) 48 h post‐transduction. Luciferase assay (B) and trypan blue exclusion viability assay (C) of hNSC‐luc^+^/GFP^+^ performed at 72 h post‐transduction. (D) In vitro BLI of hNSC‐luc^+^/GFP^+^ cells at concentrations ranging from 3 × 10^6^ to 5 × 10^2^ per well, with no luciferin and media only controls. (E) In vitro BLI of unlabelled hNSC and dead hNSC‐luc^+^/GFP^+^ (DC), with hNSC‐luc^+^/GFP^+^ cells as a positive control. (F) In vivo BLI detection of transplanted hNSC‐luc^+^/GFP^+^ in 8‐week‐old C57BL/6J mice on post‐operative day (POD) 2 after bilateral injection of 3.6 × 10^5^ hNSC‐luc^+^/GFP^+^, or unilateral injection of 1.8 × 10^5^ hNSC‐luc^+^/GFP^+^ (L: left side) with contralateral injection of 1.8 × 10^5^ DC or unlabelled hNSC transplants (*n =* 2 per group). (G) Representative POD 2 immunohistochemical (IHC) images showing the fimbria fornix target area in C57BL/6J mice, with hNSC‐luc^+^/GFP^+^ grafts expressing GFP (green) and human‐specific nuclear antibody HuNu (red), with contralateral staining of DC or unlabelled hNSC. Data presented as mean ± standard error of the mean (S.E.M.) for luciferase activity and cell viability analysed by ANOVA with Tukey's post‐test for comparisons of multiple groups. **p < .01; ****p < .0001. DC, dead cells; POD, post‐operative day

Using a molecular imaging approach, we modified hNSCs with a lentiviral vector to induce stable expression of luc and GFP reporters. GFP reporter expression was visualized in transduced hNSCs confirming transduction (Figure 1A), and expression increased with escalating MOI. For MOI selection, luciferase expression was measured in modified hNSCs, where highest activity was observed at MOI 25 (Figure 1B). There was also negligible effect on cell viability at this MOI, whereas higher MOI reduced cell viability (Figure 1C). Based on these data, a large bank of hNSC‐luc^+^/GFP^+^ transduced at MOI 25 was generated for all further experiments.

We next used optical imaging to evaluate the sensitivity and specificity of bioluminescence in hNSC‐luc^+^/GFP^+^ in vitro. Cells were serially diluted within the range of 5 × 10^2^ to 3 × 10,^6^ and luciferin was added prior to imaging for bioluminescence (Figure 1D). Bioluminescent signal was increased at greater cell densities. Media only and hNSC‐luc^+^/GFP^+^ cells without luciferin were included as negative controls and produced no signal. To assess if signal is specific to viable, labelled cells, unlabelled hNSC and dead hNSC‐luc^+^/GFP^+^ cells were also imaged and did not produce bioluminescent signal (Figure 1E).

To evaluate bioluminescence in vivo, we injected hNSC‐luc^+^/GFP^+^ bilaterally into the fimbria fornix of 8‐week‐old C57BL/6J mice and imaged on POD 2. Control groups included mice with unilateral injection of live hNSC‐luc^+^/GFP^+^ and contralateral injection of either unlabelled hNSC or dead cells. In bilaterally transplanted mice, hNSC‐luc^+^/GFP^+^ cells were detectable as substantial bilateral BLI signal (Figure 1F). In unlabelled and dead cell groups, bioluminescent signal was present unilaterally at the site of live hNSC‐luc^+^/GFP^+^ cell transplants, while the contralateral side exhibited no signal. Histological analysis confirmed that hNSC‐luc^+^/GFP^+^ grafts in postmortem mouse brain co‐localized with BLI signal (Figure 1G). Here, GFP^+^ and HuNu^+^ cells were visualized at the site of hNSC‐luc^+^/GFP^+^ transplants and BLI signal. Unlabelled hNSCs showed HuNu labelling alone, and dead cell grafts showed no HuNu or GFP signal.

Together, these initial proof‐of‐concept experiments confirmed that BLI can detect hNSC‐luc^+^/GFP^+^ cells in vitro and in vivo and indicate that bioluminescent signal is specific to viable, labelled cells, thereby validating BLI as a tool to assess in vivo survival of transplanted cells.

### 3.2 Anti‐CD4 and anti‐CD40L mAbs enable robust hNSC survival compared to traditional immunosuppression

We next used in vivo BLI tracking of hNSC survival in real time to compare immunosuppression protocols in C57BL/6J mice. For this purpose, mice were randomly assigned to the following groups: no immunosuppression, Tac/MMF, Tac/MMF/mAb or mAbs alone. hNSC‐luc^+^/GFP^+^ cells were transplanted into the fimbria fornix of the hippocampus, and mice were imaged on POD 2 and weekly until end point, approximately 7 weeks post‐transplantation. Induction Tac/MMF treatment began with daily administration starting 7 days before transplant and continuing 7 days after transplant, with Tac continuing daily until study endpoint. Induction of mAb immunosuppression began with daily administration of anti‐CD4 and anti‐CD40L starting on day of transplanted for three doses, followed by administration every 7 days until study endpoint.

**FIGURE 2:**
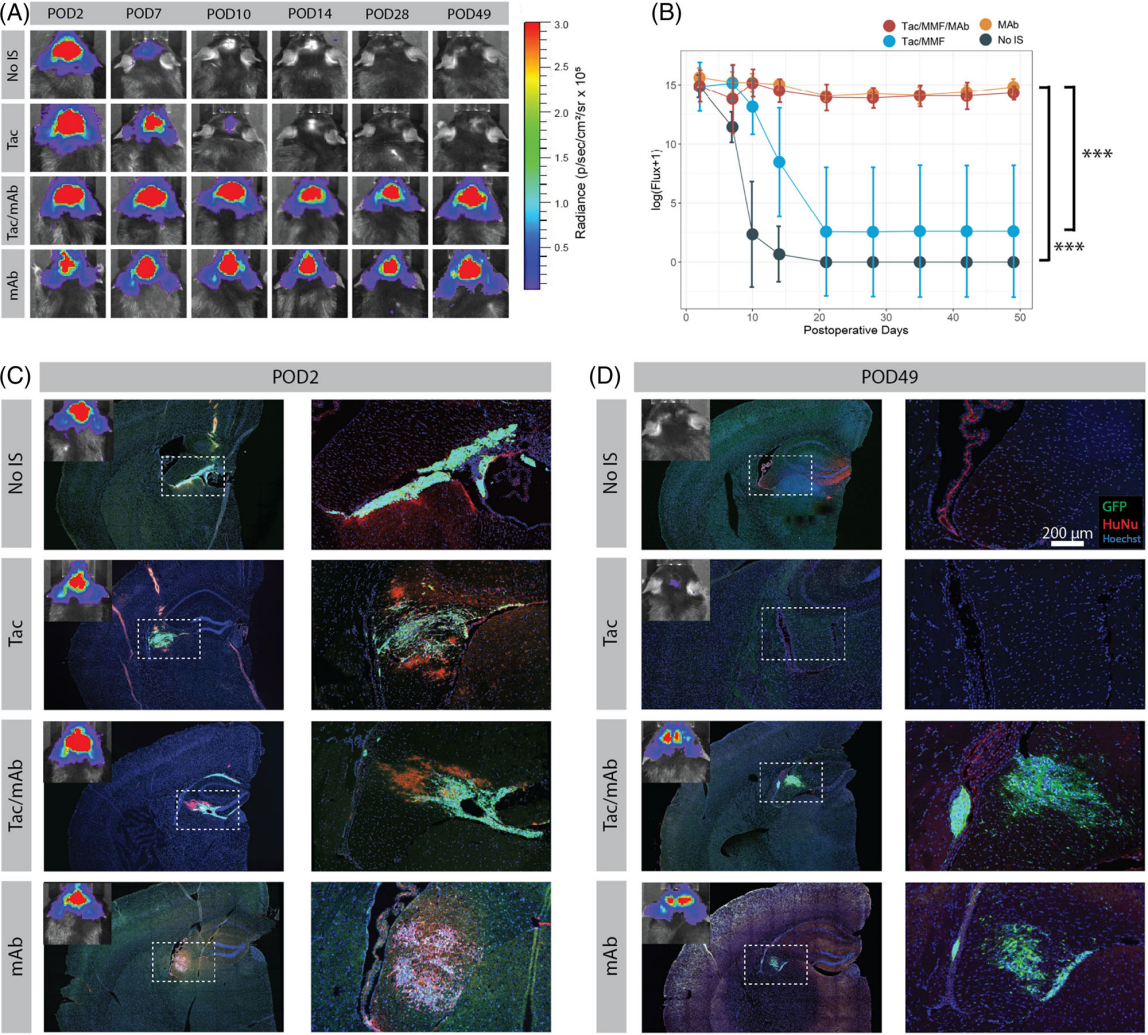
Assessment of immunosuppression protocols using bioluminescent imaging (BLI) to track transplanted hNSC graft survival. (A) Serial BLI detection of transplanted hNSC‐luc^+^/green fluorescent protein (GFP)^+^ in C57BL/6J mice (3.6 × 10^5^ total cells) receiving no immunosuppression (No IS), tacrolimus with mycophenolate mofetil (Tac/MMF), Tac/MMF in combination with mAbs against CD40L and CD4 (Tac/MMF/mAbs) or only mAb against CD40L and CD4 (mAbs). (B) BLI signal quantification for all mice at all‐time points demonstrates significant maintenance of BLI signal in mAb treated groups versus Tac/MMF alone or non‐immunosuppressed groups. (C and D) immunohistochemical (IHC) images showing GFP^+^ grafts (green) colocalized with HuNu (red) in the fimbria fornix target area at POD 2 and POD 49 (endpoint). Starting sample size: *n* = 10 in No IS group, *n* = 12 in all other groups. Subsets were euthanized for IHC at POD2 (2 from No IS group and 3 from all other groups) and POD10 (2 from No IS group and 3 from all other groups, IHC data not shown). Remaining animals were used for each BLI data point until study end (POD 49). Data presented as mean ± standard deviation (SD) for repeated BLI measures, analysed by linear mixed effects model, ***p < .001. HuNu, human nuclei; IS, immunosuppression; POD, post‐operative day

Robust BLI signal indicating graft survival was observed in all groups 2 days post‐operatively (Figure 2A). Signal loss rapidly declined after POD 7 in non‐immunosuppressed mice and at POD 14 in mice on a regimen of Tac/MMF. However, the addition of anti‐CD4 and anti‐CD40L mAbs preserved bioluminescent signal, and hence graft survival, to the terminal time point of 7 weeks.

At POD 10, graft loss appeared associated with IgG and CD4 positive staining. Animals treated with traditional Tac/MMF were compared to animals receiving combined Tac/MMF and mAb, and both demonstrated accumulation of Iba1 positive microglia at graft sites (Figure S1A), suggesting microglial reaction to hNSC grafts was not impacted by mAb. We did not detect significant CD40L at cell grafts in either group (Figure S1A). By contrast, staining for Mouse IgG as well as CD4 was only seen in Tac/MMF treated animals and was not detectable in mAb treated mice (Figure S1B). There was no detectable staining of IgM or IgA (data not shown). Higher magnification images demonstrated IgG staining on the surface of many GFP^+^ stem cells in animals treated with Tac/MMF, suggesting host‐generated antibodies were reactive to xenograft surface epitopes prior to clearance of grafts (Figure S1C). While rare inclusion bodies showed IgG staining in mAb treated animals, this was not associated with GFP‐expressing stem cells. This appears consistent with prior work describing a role for a humoural response as well as CD4^+^ T cell response to xenografts in mice.^16^ The difference in Mouse IgG detection could be a result of anti‐CD40L mAb activity in the periphery and interference with B cell Ig class switching.^40^, ^54^

Total BLI flux was quantified for all time points (Figure 2B) and was significantly increased over follow‐up time in the mAb groups as compared to non‐immunosuppressed and Tac/MMF groups (*p* < .001). No significant difference in flux was observed between Tac/MMF/mAb and mAb alone groups. Of note, in the Tac/MMF group, signal was present in only two mice from POD 14 onward, representing a survival rate of 20% (*n* = 10 total). Conversely, robust bioluminescent signal was present throughout the experiment in all mice on mAb regimens, both in the mAb alone (*n =* 7 euthanized at POD 14, *n =* 5 euthanized POD 49) and the combined Tac/MMF/mAb groups (*n =* 7 euthanized at POD 14, *n =* 5 euthanized at POD 49), representing a survival rate of 100%. This indicates that a regimen of only CD4/CD40L mAbs is sufficient to support graft survival.

To confirm the BLI results, transplanted hNSC‐luc^+^/GFP^+^ grafts were also evaluated histologically directly following POD 2 and POD 49 imaging. At the earlier POD 2 time point, bioluminescent signal corresponded with GFP^+^ cells and HuNu^+^ immunostaining in all groups, where sizeable grafts were visible in the fimbria fornix target region (Figure 2C). These cells stained strongly for the neuronal marker Nestin, with the occasional expression of astrocyte marker GFAP (Figure S1D), consistent with our previous characterization of this cell line.^40^ By POD 49, the non‐immunosuppressed and Tac/MMF groups that had lost bioluminescent signal also exhibited no human‐specific immunostaining or GFP^+^ cells (Figure 2D). Weakly GFP^+^ and HuNu^+^ debris was observed in the target areas, indicative of graft rejection. Conversely, the retention of bioluminescent signal in mAb groups corresponded with intact HuNu^+^/GFP^+^ cells in the fimbria fornix target region, demonstrating robust viability of transplanted hNSC‐luc^+^/GFP^+^ cells.

We also analysed CBC profiles on whole blood from mice that underwent hNSC transplantation and BLI (Table 1). Decreased white blood cell counts, including neutrophils, lymphocytes and monocyte counts (with preserved lymphocyte:neutrophil ratios) were observed in Tac/MMF and Tac/MMF/mAb groups as compared to non‐immunosuppressed controls, indicating a more global immunosuppression relative to non‐treated, non‐immunosuppressed controls. However, mice receiving only mAbs with hNSC transplants maintained normal CBC profiles comparable to non‐immunosuppressed mice. Eosinophil, basophil, red blood cell and platelet counts were normal for all hNSC transplant groups regardless of immunosuppressant. These data indicate that mAb immunosuppression mediates a targeted immunosuppressant activity sufficient to preserve human hNSC survival without detrimental impact to peripheral CBC profiles.

**TABLE 1:** Complete blood count (CBC) analysis. (A) Whole blood CBC analysis in C57BL/6J mice 8 weeks post‐transplantation of 360k human neural stem cell (hNSC)‐luc^+^/green fluorescent protein (GFP)^+^. CBC panel measures and data (mean ± standard deviation) for each experimental group. (B) CBC measures were compared between groups, and statistically significant comparisons are highlighted

**A. CBC results**					
**Measure**	**Normal range**	**No IS** **(*n* = 7)**	**Tac** **(*n* = 6)**	**Tac mAb** **(*n* = 7)**	**mAb** **(*n* = 7)**
White blood cell count ‐ WBC (10^3^/μl)	1.8–10.7	3.123 ± .5019	1.007 ± .5417	1.194 ± .7348	3.28 ± 1.469
Neutrophil count ‐ NE (10^3^/μl)	.1–2.4	.8957 ± .5754	.2067 ± .1122	.2643 ± .1295	.6986 ± .3076
Lymphocyte count ‐ LY (10^3^/μl)	.9–9.3	2.119 ± .4143	.755 ± .4117	.8714 ± .5934	2.471 ± 1.165
Monocyte count ‐ MO (10^3^/μl)	.0–.4	.09143 ± .02545	.03 ± .0228	.04429 ± .02637	.09429 ± .07829
Eosinophil count ‐ EO (10^3^/μl)	.0–.2	.01 ± .005774	.008333 ± .007528	.008571 ± .006901	.01429 ± .01272
Basophil count ‐ BA (10^3^/μl)	.0–.2	.004286 ± .005345	.003333 ± .008165	.004286 ± .005345	.002857 ± .00488
Red blood cell count ‐ RBC (10^6^/μl)	6.36–9.42	8.579 ± .31	8.478 ± .8597	9.18 ± .7665	9.134 ± .4404
Hemoglobin ‐ HB (g/dl)	11.0–15.1	11.69 ± .3891	10.97 ± 1.234	11.91 ± .4634	11.73 ± .6317
Hematocrit ‐ HCT (%)	35.1–45.4	37.01 ± 1.471	35.63 ± 3.976	38.17 ± 1.608	39.47 ± 2.391
Mean cell volume ‐ MCV (fL)	45.4–60.3	43.14 ± .5442	42 ± .6663	41.71 ± 1.875	43.2 ± .8794
Mean cell hemoglobin ‐ MCH (pg)	14.1–19.3	13.64 ± .1902	12.9 ± .2098	13.01 ± .7151	12.83 ± .4751
MCH concentrate ‐ MCHC (g/dl)	30.2–34.2	31.59 ± .3805	30.78 ± .4167	31.21 ± 1.014	29.73 ± .9759
Red cell distribution width ‐ RDW (%)	12.4–27.0	17.74 ± .4894	17.18 ± .7305	18.24 ± 1.247	18.04 ± .4036
Platelet count ‐PLT (10^3^/μl)	592—2972	439.9 ± 197.1	377.3 ± 250.2	472.3 ± 249.2	662.3 ± 105.1
Mean platelet volume ‐ MPV (fL)	5.0–20.0	4.543 ± .4198	4.717 ± .6401	4.514 ± .7988	4.271 ± .1799

**B. CBC statistics**							
**Group comparison**	**WBC**	**NE**	**LY**	**MO**	**EO**	**BA**	**RBC**
No IS versus Tac	****	***	***	*	ns	ns	ns
No IS versus Tac MAb	***	***	**	*	ns	ns	ns
No IS versus MAb	ns	ns	ns	ns	ns	ns	ns
Tac versus Tac MAb	ns	ns	ns	ns	Ns	ns	*
Tac versus MAb	****	**	****	**	Ns	ns	ns
Tac MAb versus MAb	****	*	****	*	Ns	ns	ns

* Significant (*p* < .0332) by one‐way ANOVA.

** Significant (*p* < .0021) by one‐way ANOVA.

*** Significant (*p* < .0001) by one‐way ANOVA.

Together, these data show that a regimen of anti‐CD4 and anti‐CD40L mAbs extends the survival of intracranial human stem cell transplants through at least 7 weeks in C57BL/6J mice and does not negatively impact CBCs compared to the conventionally used immunosuppressive agents Tac/MMF, offering an enhanced immunosuppression method for xenografts in mice.

### 3.3 Both anti‐CD4 and anti‐CD40L mAbs are required for transplanted hNSC graft survival

**FIGURE 3:**
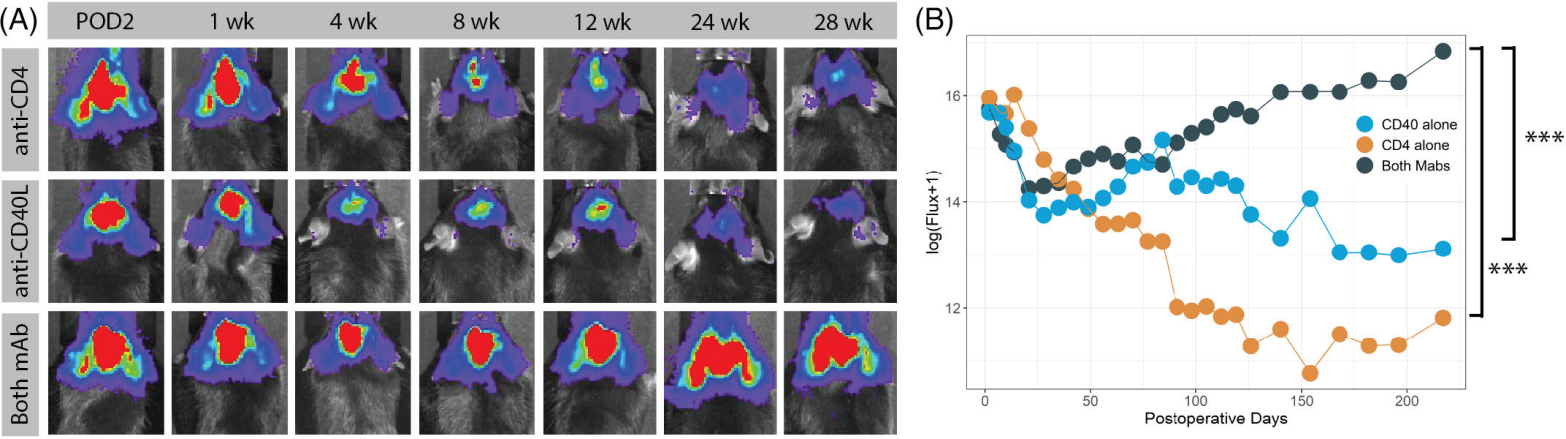
Both anti‐CD4 and anti‐CD40L mAbs are required for human neural stem cell (hNSC) graft survival. (A) Serial bioluminescent imaging (BLI) detection of transplanted hNSC‐luc^+^/green fluorescent protein (GFP)^+^ in C57BL/6J mice (3.6 × 10^5^ total cells) receiving anti‐CD4 mAb, anti‐CD40L mAb or both mAbs. (B) BLI signal quantification for all mice at all‐time points. Dual mAb treatment resulted in significantly greater persistence of BLI signal. Sample size: *n* = 5 animals per group. Data presented as mean for BLI measures, error bars omitted for clarity, analysed by linear mixed effects model, ***p < .001. POD, post‐operative day, wk = week

Although anti‐CD4 or anti‐CD40L mAbs have been used in combination with other agents to promote the short‐term acceptance of transplanted organ and cell grafts in mice,^33^ ^,^ ^55^, ^56^ they have not well studied individually in the context of CNS xenograft transplantation. Therefore, we next questioned if suppression of both CD4^+^ and CD40L^+^ cells is required, or if administration of a single mAb is sufficient for long‐term transplanted hNSC graft survival. To determine this, hNSC‐luc^+^/GFP^+^ cells were transplanted intracranially to C57BL/6J mice on a regimen of anti‐CD4 mAb alone, anti‐CD40L mAb alone, or a combination of both anti‐CD4 and anti‐CD40L mAbs. Induction mAb treatment began 1 day prior to transplant for 4 daily doses, and continued every 7 days until study endpoint. Serial BLI imaging was again used to track hNSC survival over time and was performed beginning on POD 2 and weekly or biweekly until end point, approximately 28 weeks post‐transplantation (Figure 3A). Mice receiving both mAb retained BLI signal strength through 28 weeks, indicative of hNSC graft survival. However, similar graft survival was not maintained in the anti‐CD4 alone and anti‐CD40L alone groups. This was also reflected in the signal quantification (Figure 3B), where total flux was similar for all groups at POD 2 then separated longitudinally, with the dual‐mAb group retaining significantly increased signal throughout the 28‐week time span as compared to anti‐CD4 alone (*p* < .001) or anti‐CD40L alone (*p* < .001).

To confirm and characterize the effect of mAb immunosuppression on the host immune response, we also assessed CD4^+^ cells and soluble CD40L levels in peripheral blood. Here, a separate cohort of animals received anti‐CD4 alone, anti‐CD40L alone or dual mAb therapy on an induction schedule of four daily doses of mAb. Flow cytometry for CD4^+^ cells was performed on peripheral whole blood collected 1 week after the induction regimen of mAb. Mice in the anti‐CD4 alone and dual mAbs groups displayed depletion of CD4^+^ cells, relative to mice that were treated with anti‐CD40L alone and untreated controls (Figure S2A). Numbers of CD40L^+^ T cells were poorly detected by flow cytometry even in untreated animals (Figure S2B) likely due to the transient nature of surface CD40L expression with T cell activation.^57^, ^58^ Therefore, serum was taken from animals after completion of BLI studies above and analysed for soluble CD40L. ELISA analysis of soluble CD40L levels in serum collected at experimental endpoint (28 weeks) for hNSC‐transplanted animals, only mice treated with anti‐CD40L, either alone or in combination with anti‐CD4 mAb, displayed depletion of soluble CD40L, relative to controls (Figure S2C).

### 3.4 CD4 and CD40L mAb regimen is effective at multiple cell doses and in a transgenic mouse model

**FIGURE 4:**
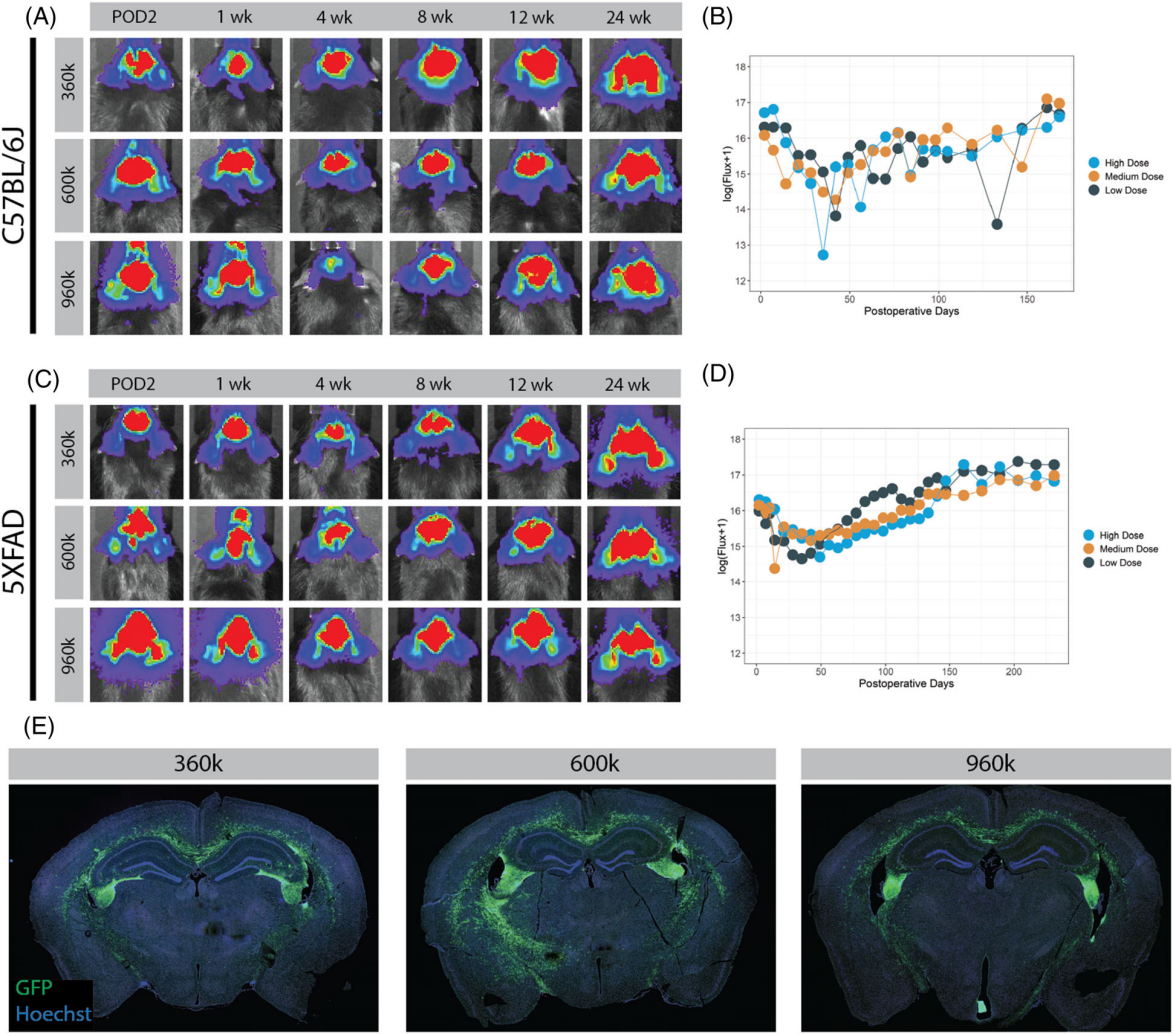
Long‐term bioluminescent imaging (BLI) tracking of transplanted human neural stem cell (hNSC) in C57BL/6J and 5XFAD. Serial BLI detection and signal quantification of 3.6 × 10^5^, 6.0 × 10^5^ or 9.6 × 10^5^ hNSC‐luc^+^/green fluorescent protein (GFP)^+^ cells transplanted in C57BL/6J mice (A and B) and 5XFAD Alzheimer's disease mice (C and D) on a dual mAb immunosuppression protocol of anti‐CD4 and anti‐CD40L. No biologically relevant statistical differences in BLI flux are seen between cell dose groups. (E) Representative immunohistochemical (IHC) images demonstrate GFP^+^ hNSC‐luc^+^/GFP^+^ grafts in the fimbria fornix target area at endpoint in C57BL/6J mice. Sample size: *n* = 5 animals per treatment dose (*n* = 15 animals for C57BL/6J and another 15 for 5XFAD). Data presented as mean for BLI measures, error bars omitted for clarity, analysed by linear mixed effects model. POD, post‐operative day

After demonstrating that dual mAb‐based immunosuppression is more effective than Tac/MMF‐based regimens or each mAb alone, we next investigated this method of immunosuppression using multiple cell doses in a transgenic mouse model. Induction of immunosuppression by daily dual mAb therapy was again performed starting 1 day prior to transplant for four doses, followed by maintenance dosing every 7 days until study endpoint. Long‐term graft survival was initially assessed in 8‐week‐old C57BL/6J mice. Multiple doses of hNSC‐luc^+^/GFP^+^ cells were transplanted bilaterally into the fimbria fornix at increasing doses of 3.6 × 10^5^ (low dose), 6.0 × 10^5^ (medium dose) and 9.6 × 10^5^ (high dose) total cells, and longitudinal BLI was performed to track graft survival. Robust BLI signal indicative of hNSC graft survival was present up to the study endpoint 24 weeks post‐transplantation (Figure 4A). While a statistical difference in the BLI signal was seen in comparing the medium and the low dose cell groups (*p* = 0.035), this difference was thought to be attributable to outlier BLI flux timepoints in the low‐ and high‐dose groups and deemed not biologically meaningful (Figure 4B). Functionally, there was no difference in BLI signal between administered cell doses.

Using an identical experimental design, we assessed mAb immunosuppression in 8‐week‐old 5XFAD mice, a commonly used model of Alzheimer's disease. Escalating doses of 3.6 × 10,^5^ 6.0 × 10^5^ and 9.6×10^5^ total cells were transplanted bilaterally into the fimbria fornix, and weekly BLI was again performed to track graft survival over time until the study termination at approximately 8 months post‐transplantation (data concordant with C57BL/6J mice up to 24 weeks presented here). Here, similar long‐term hNSC graft survival was achieved in 5XFAD mice, where robust BLI signal was present in all three cell dose groups until study endpoint (Figure 4C). Again, while statistical differences were noted between groups in effect of treatment dose over time (low‐ vs. medium‐dose, *p* = .013; low‐ vs. high‐dose, *p* < .001), stratification and correlation of BLI signal to treatment group could not be consistently identified (Figure 4D).

To address the question of differential hNSC survival rates between strains, we performed a comparison of within‐individual percent change of log (Flux+1) between first measurement to post‐operative day 161 (latest shared time point) using Wilcoxon‐Mann‐Whitney tests. There were no significant differences between C57BL/6J and 5XFAD animals in low dose (*p* = .73), medium dose (*p* = .56) or high dose (*p* = .53) groups, or even all groups combined (*p* = .61).

BLI data were confirmed histologically, where GFP^+^ hNSC grafts were visible in the fimbria fornix target of C57BL/6J (Figrue 4E). Whole brain imaging revealed significant migration of hNSCs from the site of transplantation at the fimbria fornix throughout the white matter tracts, including the corpus callosum. This migratory action may account for the apparent BLI signal increase over the long‐term period of 6 months. Histopathologic toxicity screening (blinded to treatment group) was performed at 6 months after transplant of C57BL/6J animals receiving low, medium and high dose hNSC grafts and dual mAb immunosuppression. This revealed ‘no findings suggestive of treatment‐related toxic effect in the experimental group, based on hematologic, gross pathology and histological evaluation of representative organs’ (Table S1, full report available upon request).

We were further able to confirm in 5XFAD mice that the known behavioural deficit in hippocampal‐based memory tasks was not affected by injection regimen or mAb treatment. Delayed learning curve during training period for Morris Water Maze was noted in all 5XFAD animals as compared to WT controls, regardless of whether animals received mAb, saline injections alone or no treatment (Figure S3A). Further, there was no difference in 5XFAD groups during probe trials as measured by time spent exploring the target quadrant (Figure S3B). Together, these data demonstrate that dual mAb immunosuppression supports robust hNSC survival at multiple cell doses for up to 6 months in C57BL/6J mice and up to 8 months in the 5XFAD transgenic model of Alzheimer's disease.

## 4 DISCUSSION

A significant obstacle facing the widespread adoption of stem cell therapy for neurological diseases is transplant rejection by the host immune system and eventual graft failure.^16^, ^59^ As progress is made in developing cell‐based treatments, reliable methods to overcome host immune rejection are of paramount importance. In the present research, we show that an immunosuppressive regimen of depleting anti‐CD4 and anti‐CD40L mAbs results in robust long‐term persistence of hNSC grafts, superior to the traditional agents Tac/MMF. Cell survival requires both antibodies for maximal efficacy, persists across escalating stem cell doses and is applicable to disease models. The mAb regimen is also more effective and less toxic than conventional immunosuppressive agents. Overall, this study supports the use of dual mAbs as an alternative immunosuppression method for study of human stem cell therapeutics in preclinical animal disease models.

For cell‐based therapy to succeed in clinical translation, transplanted cells must survive. In allogenic transplant paradigms, a variety of strategies have been pursued to achieve this. Prior work has utilized mesenchymal stem cells, given the relative ease of sourcing from bone marrow and early thought that mesenchymal stem cells could avoid immune rejection.^60^ However, follow‐up studies demonstrated that allogenic mesenchymal stem cell transplants still elicited an immune response and graft rejection in the ‘immune privileged’ CNS.^61^, ^62^, ^63^ Furthermore, while mesenchymal stem cells can be transdifferentiated to neuron‐like cells in vitro, their ability to recapitulate neurons in vivo after transplantation has been less studied.^64^, ^65^ Alternatively, the advent of induced pluripotent stem cell technology brings the promise of autologous cell transplants, but currently these approaches are labour‐ and cost‐intensive and likely to be prohibitive for large clinical trials and population scale therapy. Therefore, as a more practical paradigm, hNSC lines represent a promising ‘off the shelf’ therapeutic option in neurologic disorders given the capacity for large‐scale expansion and commitment to neuroglial differentiation. These advantages have led to many early phase trials utilizing hNSC lines.^7^, ^8^, ^9^, ^10^, ^11^

In preclinical testing for future therapies, hNSC studies encounter the added barrier of requiring xenograft animal models to demonstrate efficacy even prior to invoking an allograft paradigm in human trials. A number of studies use immunodeficient or humanized animal strains,^66^ yet this broad approach removes a critical contribution of the immune system to underlying disease pathophysiology of the animal model.^67^ Many groups utilize traditional pharmaceutical‐based immunosuppression based on solid organ transplant protocols. As an example, preclinical studies supporting Phase 1 and 2 clinical trials of a human spinal cord stem cell line in amyotrophic lateral sclerosis utilized Tac/MMF.^68^, ^69^, ^70^, ^71^ Yet these drugs proved to be insufficient in long‐term xenograft models. This is consistent with our own published work in various mouse models where immunosuppression with a combination of Tac/MMF resulted in low survival rates and graft clearance within 8 weeks.^41^, ^47^ Furthermore, long‐term use of these drugs may directly impact the fidelity of the underlying disease model,^72^ ^,^ ^73^ and prolonged use of these drugs is associated with toxic side effects, including elevated risk of infections, diabetes, hypertension, cardiovascular disease, nephrotoxicity and neurotoxicity.^74^ Therefore, a robust immunosuppressive regimen that ensures hNSC survival without impacting the model phenotype is critically needed.

In the current study, dual mAb therapy resulted in cell survival in 100% of transplanted animals for at least 6–8 months in both C57BL/6J mice and an aggressive model of AD, with no disruption in peripheral blood profiles or other organ toxicity. There was also no detrimental impact to the behavioural phenotype under study. We expect this targeted approach is a major advance enabling the study of a wide range of stem cell therapies in the CNS. CD4^+^ T cells play a central role in cytotoxic rejection of transplanted cells, particularly more mature or differentiated cells that up‐regulate major histocompatibility complex class I and II in the setting of local inflammation.^21^, ^24^, ^75^, ^76^ Therefore, depleting CD4^+^ T cells targets a fundamental immune rejection mechanism. CD40L also appears to be a critical component of the immune recognition and rejection of stem cell xenografts, as demonstrated by our data showing mAbs targeting both CD4 and CD40L are required for optimal cell survival. We did not detect significant changes in membrane‐bound CD40L with peripheral or infiltrating T cells, and hypothesize that CD40L plays a role in antigen presentation in the periphery for activation of stem cell‐responsive immune cells.^77^, ^78^, ^79^ However, constitutive expression of CD40L in licensed T cells within rejecting grafts appears unnecessary for immune clearance, at least in the CNS.^80^ Our observed CD4 and mouse IgG staining at the xenograft site are likely downstream effects of CD40L activation, which is curtailed with dual mAb therapy. At the same time, in contrast to broad pharmacologic immunosuppression, this targeted approach appears to minimize collateral adverse effects. This is evidenced by reduced alterations in CBC profiles of mAb‐treated animals versus those receiving Tac/MMF, as well as lack of histopathologic toxicity. A prior study by Ager et al.^81^ reported survival of CNS xenografts for up to 6 weeks in two AD mouse models utilizing mAbs targeting LFA‐1, CD40 and CTLA‐4, representing an improvement over traditional immunosuppressant drugs. A number of alternate mAb‐based approaches have been pursued targeting the T cell costimulatory mechanism, including targeting CD4 alone,^20^ ^,^ ^31^, ^82^ CD2/T‐cell receptor αβ,^83^ CD25,^84^ IL‐2R,^85^ CTLA‐4/MR1^86^ and B7/LFA‐1,^80^ ^,^ ^87^ although these studies did not interrogate cell survival for more than 4–18 weeks. Here, our data demonstrate that anti‐CD4 mAb alone still results in a significant degree of graft loss over the long‐term, while dual mAbs produces robust graft survival for more than 28 weeks.

Although we found statistical differences in BLI signal over time with varying cell doses in C57BL/6 and 5XFAD mice, variability in BLI quantification curves precludes conclusions on biological relevance. Some variability in BLI signal is likely related to migration and spread of luminescent cells along white matter tracts in the brain. Furthermore, it is possible that a maximal cell dose was reached, and additional cell survival at higher cell number/concentration could not be supported in the transplant site.^88^ We are performing additional dosing studies using an unmodified hNSC cell line to further investigate and identify the maximal tolerated dose in AD mice. As this approach is extended to other disease models, the preservation of therapeutic benefit as well as underlying animal model phenotype will need to be validated for each cell line/model in the presence of dual mAb‐based immunosuppression.

The approach presented here is readily translatable to clinical trials of stem cell therapy. Therapies targeting CD4 (clenoliximab, keliximab, zanolimumab) and CD40(teneliximab) are pending or already under investigation in human trials.^36^, ^89^, ^90^ Still, several obstacles remain in clinical application of stem cell therapies for neurological diseases. Although we saw no behavioural difference with mAb treatment in our AD mouse model, given the complex interaction of the nervous system and the immune system, global impacts of chronic mAb treatment especially in humans will need further study. Furthermore, while we modified transplanted cells to express luc/GFP for in vivo BLI, alternate methods will be required for non‐invasive cell tracking in humans. Current techniques, such as superparamagnetic iron oxide labelling or use of radiotracers, can become diluted over time and are also limited by the inability to distinguish between live cells versus phagocytosed dead cell debris.^91^, ^92^ Thus, future studies must also address a viable cell tracking approach in humans.

The present study has a number of limitations and is focused on optimizing survival of hNSC grafts in murine models, including 5XFAD model of Alzheimer's disease. Our data demonstrate cell survival in an AD mouse model; still, many questions regarding therapeutic benefit of hNSCs and potential mechanisms of action remain to be answered in ongoing larger‐scale preclinical studies. We show that dual mAb therapy and the anxiety from repeated handling/injections do not adversely affect the fidelity of the cognitive deficits seen in this model.^42^ However, these studies were not designed or powered to assess efficacy of hNSCs as a therapy for Alzheimer's disease. Our studies with mAb immunosuppression are limited to a single human NSC line that is currently under therapeutic development for AD as part of the NIA Alzheimer's Drug Development Program (5U01AG057562). Larger‐scale efficacy studies are ongoing, enabled by discoveries presented here. Feasibility must also be established for other cell types of interest, such as those derived from iPS or ES cells. We also altered our cell line to express luciferin and GFP. We therefore cannot conclude that graft survival was not purely mediated by immune tolerance to expression of luciferin or GFP specifically, as opposed to other human epitopes on the transplanted cells.

While we demonstrated superiority of dual mAb treatment to traditional immunosuppression, it remains unclear if maintenance mAb treatments are required indefinitely. Ongoing weekly antibody infusions would be challenging for patients, although infusion in human subjects could be extended based on antibody pharmacokinetics^93^ or using techniques to extend antibody half‐life^94^ Animal data suggest cessation of immunosuppression results in graft loss even at later time points,^32^ although scant human data show persistence of some cells after a limited course of immunosuppression.^95^

As mAb mature in the clinical realm, their use and potential drawbacks will require long‐term study. Although current solid organ/bone marrow transplant patients carry elevated risk of infection and medication side effets with current immunosuppressants, ongoing depletion of CD4 and CD40L in humans is also likely to carry some risk. While we did not see significant adverse events in our well‐controlled animal population, infection risks and adverse effects of sustained CD4/CD40L depletion will need to be accounted for and compared to traditional regimens in any future application in humans.^96^, ^97^ Given the mechanism of targeting costimulatory activation in the presence of transplanted stem cell antigen presentation, it is theoretically possible host tolerance could be induced or that T cells could be rendered anergic to transplanted antigens.^80^, ^87^ In future studies, if a limited course of mAb therapy could be defined that preserves stem cell viability, side effect profiles and translational potential could be improved even further.

The results presented here are important for several reasons. Establishing an efficacious targeted immunosuppression treatment that ensures hNSC graft survival enables preclinical investigation and translation of cell‐based therapeutics in the CNS. We have demonstrated durable xenograft cell survival over an extended period of at least 28 weeks, longer than other reported studies.^31^, ^32^ This informs future preclinical studies that may require longer timelines and serves as a guideline for cellular therapies that translate to early clinical trials. As the advantages of stem cell‐based treatment paradigms become increasingly utilized, the robust immunosuppression regimen outlined here will optimize long‐term rates of success.

## 5 CONCLUSIONS

mAbs (anti‐CD4 and anti‐CD40L) enable long‐term (>6 months) survival of hNSCs grafted into brains of laboratory mice and a mouse model of Alzheimer's disease. This facilitates robust preclinical study of cell‐based therapy for CNS disorders and fast‐tracks translation of stem cell therapy to early phase clinical trials.

## CONFLICT OF INTEREST

The authors report no conflict of interest.

## Supporting information


**FIGURE S1. Characterization of hNSC grafts and mediators of graft rejection**. Phenotype of grafted hNSCs was confirmed, and assessments of immunologic mediators of graft rejection were performed using immunohistochemical (IHC) staining. Select immune populations were unaffected by mAb treatment (**A**). This included Iba1^+^ microglia, seen to aggregate in grafts of animals receiving Tac/MMF and also in animals receiving Tac/MMF. No significant infiltrating CD40L^+^ cells were noted within any graft sites. By contrast, differences were seen in other immune markers (**B**). Presence of IgG^+^ and CD4^+^ cells in the graft site was noted in animals receiving Tac/MMF but absent in those receiving Tac/MMF and mAb (white dashed outline). Representative high magnification images demonstrate IgG staining associated with cell surface of GFP^+^ stem cells (**C**, white arrows areas of IgG staining on GFP^+^ hNSCs). In characterizing differentiation, IHC at POD2 shows GFP positive hNSC‐luc^+^/GFP^+^ grafts with largely concordant Nestin staining, confirming that the vast majority of cells are committed to neuronal fates (**D**). Very minimal staining for astrocyte marker glial fibrillary acidic protein (GFAP) was associated with hNSC grafts. POD, post‐operative day.

**FIGURE S2. In vivo validation of anti‐CD4 and anti‐CD40L mAb treatment in C57BL/6J**. Whole blood flow cytometry analysis as a percentage of all peripheral lymphocytes in C57BL/6J mice that received no treatment (NT), anti‐CD4 mAb, anti‐CD40L mAb or both mAbs. Specific depletion of CD4^+^ cells is noted in animals receiving anti‐CD4 antibody (**A**). No significant detectable membrane‐bound CD40L was noted in any group by flow cytometry (**B**). ELISA quantification of serum soluble CD40L levels showed specific depletion in animals receiving anti‐CD40L mAb (**C**). Sample size (Flow cytometry/ELISA): NT *n =* 14/4; anti‐CD4 mAb *n =* 6/7; anti‐CD40L mAb *n =* 6/5; both mAbs *n =* 12/8. Data presented as mean ± standard error of the mean (SEM) analysed by one‐way ANOVA with Tukey's post‐test for comparisons of multiple groups. *p < .05; **p < .01; ***p < .001; ****p < .0001.

**FIGURE S3. Performance in the Morris water maze is not affected in mice treated with mAbs**. Mice were examined in the Morris water maze to assess the impact of serial intraperitoneal injections and chronic CD4/CD40L mAb treatment. These groups included WT mice with no treatment as well as 5XFAD mice with no treatment, 5XFAD mice that received biweekly injections of saline or CD4/CD40L mAb. Intraperitoneal injections began 4 weeks prior to initiation of behavioural testing. **(A)** Mice were trained for 12 days (D1‐D12 on x‐axis) with four trials a day. During each trial mice were placed at random locations around the edge of the pool and allowed to swim for 60 s or until they found the platform, which was hidden just below the surface of the water. The latency to locate the hidden platform significantly decreased across training days in all groups regardless of genotype or treatment (*p* < .0001, repeated measures ANOVA main effect of training; no main effect of genotype/treatment). **(B)** To evaluate long‐term memory (24 h), probe trials were conducted prior to start of training on day 4 and 24 h after the end of training (day 13). During the probe trials, the platform was removed, and mice were allowed to swim for a total of 60 s. The percentage of time that mice spent searching in the quadrant of the pool where the platform was previously located (target quadrant) was calculated as a measure of spatial memory. During the probe trial conducted on day 4 (first arrow in panel A), WT mice exhibited a selective search strategy, spending significantly more time in the target quadrant (*p < .05, 1‐sample *t*‐test against chance [25%: dashed line in figure]) whereas the 5XFAD mice exhibited a random search strategy regardless of treatment. During the probe trial carried out on day 13, all mice exhibited a selective search strategy (*p < .05, 1‐sample *t*‐test against chance [25%: dashed line in figure]). Furthermore, performance in the Morris water maze appeared to be unaffected by serial intra‐peritoneal injections or chronic CD4/CD40L mAb treatment. To control for motivation, swimming ability, and sensory perception, mice were run in the visible‐platform version of the water maze on the day following the final probe trial (VP in panel A). In this version, a distinct local cue (a flag) was fixed to the center of the hidden platform. Mice were given four visible‐platform trials with a maximum of 60 s per trial. Sample size: *n* = 8 animals in WT No Tx, FAD No Tx, and FAD mAb groups, *n* = 7 animals in FAD saline group. There were no significant differences in the latency to locate the hidden platform between any of the groups. Data are presented as mean ± SEM WT = wild type, Tx = treatment

TABLE S1: Histopathologic toxicity screen on stem cell injected C57BL/6J mice. Histopathologic analysis was performed to screen for toxicity from stem cell treatment (Group A = 3.6 × 10^5^ cells, Group B/C = 6.0 × 10^5^ cells, Group D = 9.6 × 10^5^ cells) and dual mAb immunosuppression at 6 months post‐hNSC transplantation. In examined tissues, no significant findings were noted (‐) except for occasional findings in liver of focal mononuclear infiltration or centrilobular necrosis (^†^background findings in mice) or portal vein hypoplasia/hepatic arteriolar duplication (^‡^background finding in C57BL/6J mice).
