# Supplementary figures and images for: Monoclonal antibody‐mediated immunosuppression enables long‐term survival of transplanted human neural stem cells in mouse brain
Source: Clin Transl Med. 2022 Sep 13;12(9):e1046. doi: 10.1002/ctm2.1046 (PMC9471059; doi:10.1002/ctm2.1046)

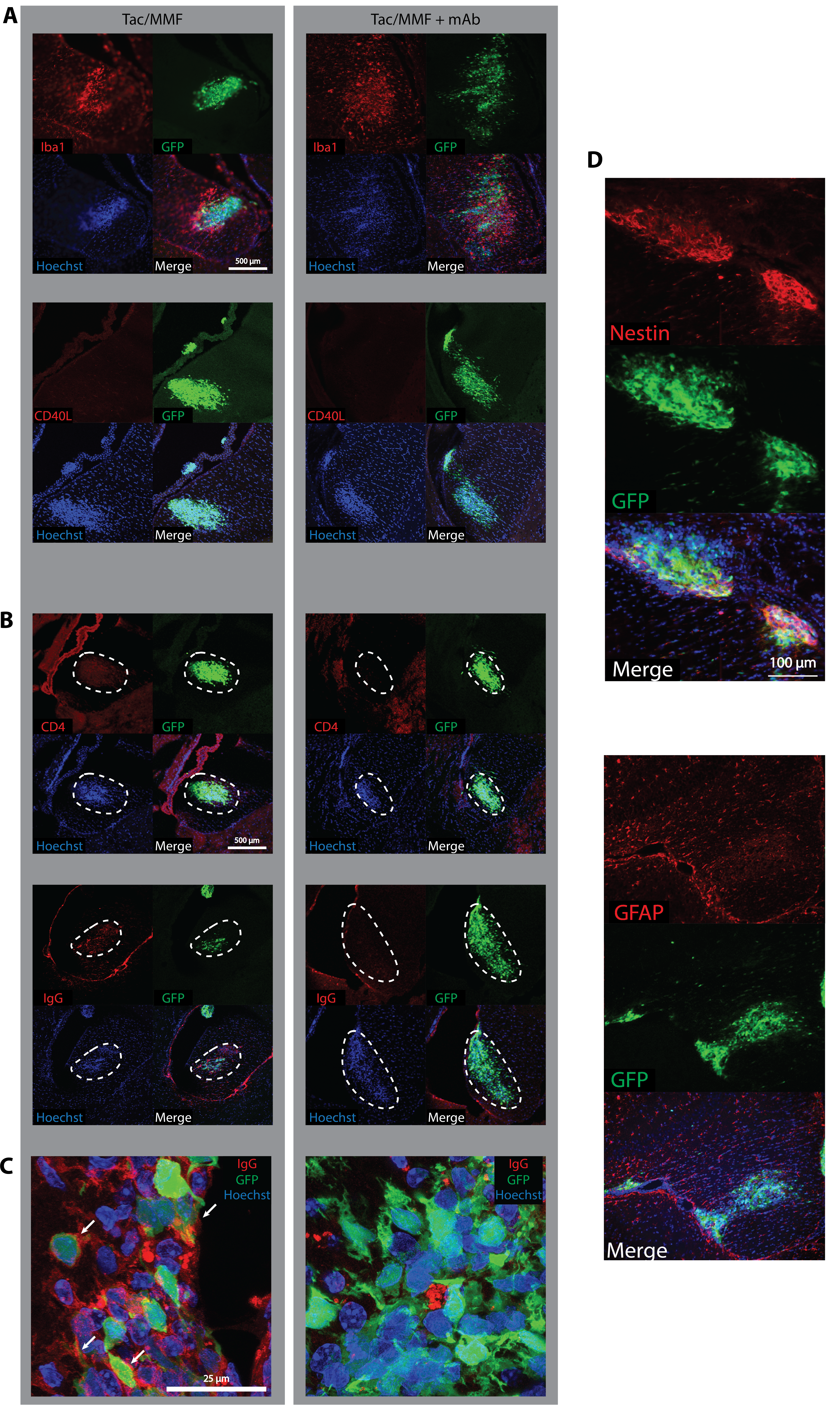

Supplement: Supplementary file 1 — FIGURE S1. Characterization of hNSC grafts and mediators of graft rejection. Phenotype of grafted hNSCs was confirmed, and assessments of immunologic mediators of graft rejection were performed using immunohistochemical (IHC) staining. Select immune populations were unaffected by mAb treatment (A). This included Iba1+ microglia, seen to aggregate in grafts of animals receiving Tac/MMF and also in animals receiving Tac/MMF. No significant infiltrating CD40L+ cells were noted within any graft sites. By contrast, differences were seen in other immune markers (B). Presence of IgG+ and CD4+ cells in the graft site was noted in animals receiving Tac/MMF but absent in those receiving Tac/MMF and mAb (white dashed outline). Representative high magnification images demonstrate IgG staining associated with cell surface of GFP+ stem cells (C, white arrows areas of IgG staining on GFP+ hNSCs). In characterizing differentiation, IHC at POD2 shows GFP positive hNSC‐luc+/GFP+ grafts with largely concordant Nestin staining, confirming that the vast majority of cells are committed to neuronal fates (D). Very minimal staining for astrocyte marker glial fibrillary acidic protein (GFAP) was associated with hNSC grafts. POD, post‐operative day. [file CTM2-12-e1046-s003.png]

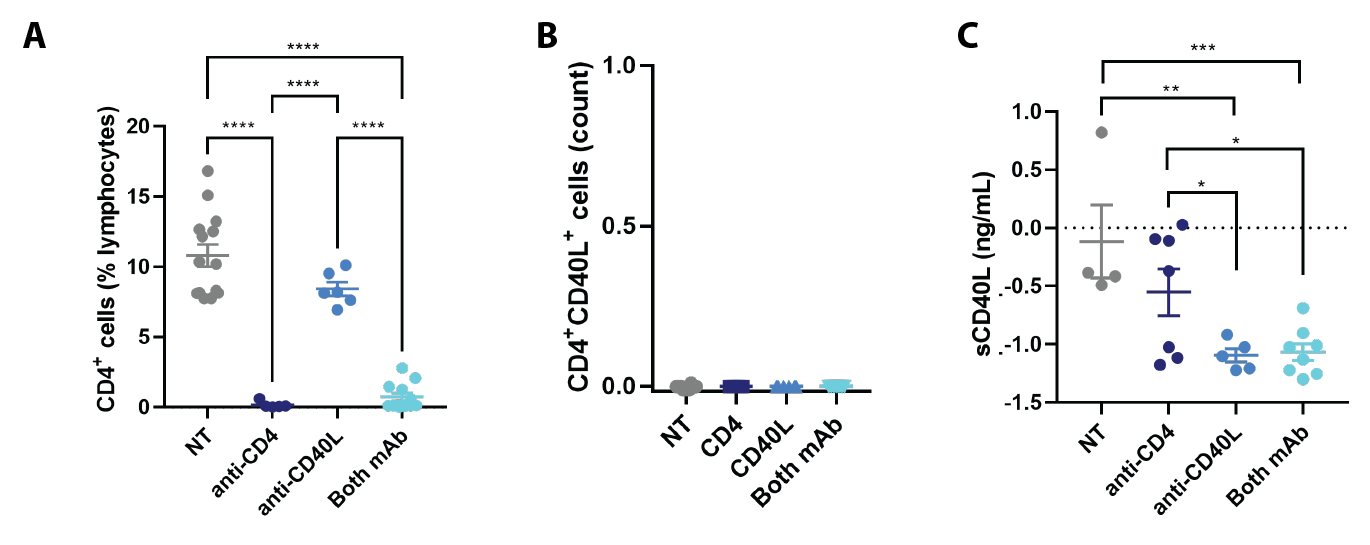

Supplement: Supplementary file 2 — FIGURE S2. In vivo validation of anti‐CD4 and anti‐CD40L mAb treatment in C57BL/6J. Whole blood flow cytometry analysis as a percentage of all peripheral lymphocytes in C57BL/6J mice that received no treatment (NT), anti‐CD4 mAb, anti‐CD40L mAb or both mAbs. Specific depletion of CD4+ cells is noted in animals receiving anti‐CD4 antibody (A). No significant detectable membrane‐bound CD40L was noted in any group by flow cytometry (B). ELISA quantification of serum soluble CD40L levels showed specific depletion in animals receiving anti‐CD40L mAb (C). Sample size (Flow cytometry/ELISA): NT n = 14/4; anti‐CD4 mAb n = 6/7; anti‐CD40L mAb n = 6/5; both mAbs n = 12/8. Data presented as mean ± standard error of the mean (SEM) analysed by one‐way ANOVA with Tukey's post‐test for comparisons of multiple groups. *p < .05; **p < .01; ***p < .001; ****p < .0001. [file CTM2-12-e1046-s001.png]

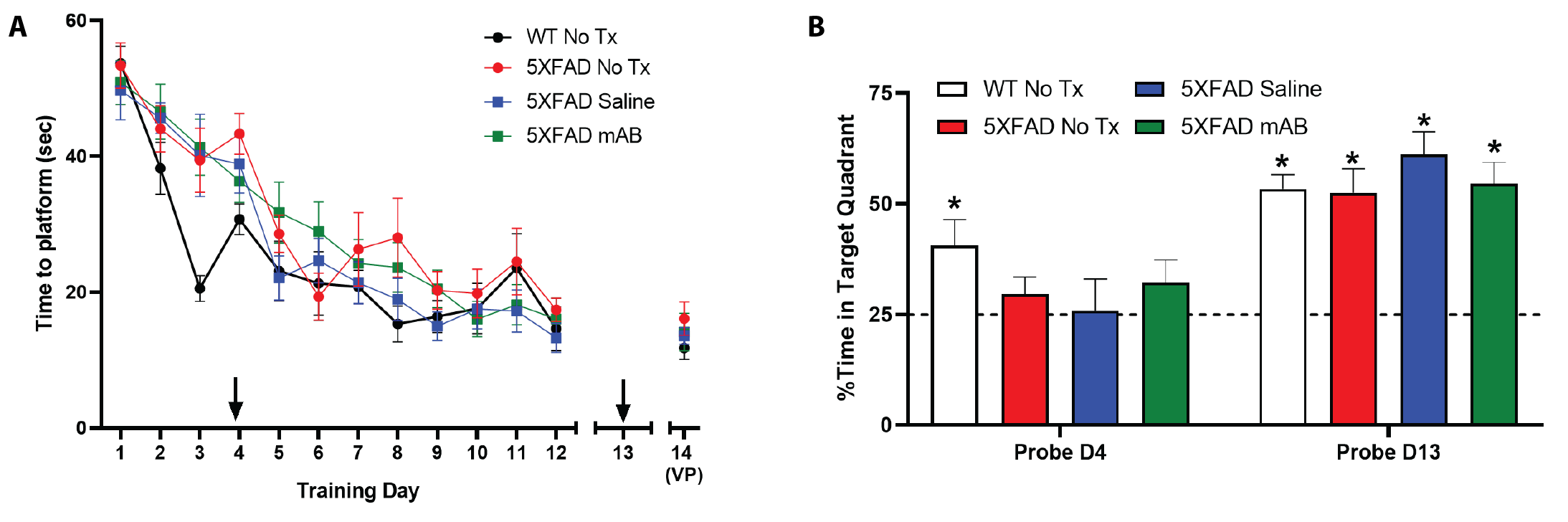

Supplement: Supplementary file 3 — FIGURE S3. Performance in the Morris water maze is not affected in mice treated with mAbs. Mice were examined in the Morris water maze to assess the impact of serial intraperitoneal injections and chronic CD4/CD40L mAb treatment. These groups included WT mice with no treatment as well as 5XFAD mice with no treatment, 5XFAD mice that received biweekly injections of saline or CD4/CD40L mAb. Intraperitoneal injections began 4 weeks prior to initiation of behavioural testing. (A) Mice were trained for 12 days (D1‐D12 on x‐axis) with four trials a day. During each trial mice were placed at random locations around the edge of the pool and allowed to swim for 60 s or until they found the platform, which was hidden just below the surface of the water. The latency to locate the hidden platform significantly decreased across training days in all groups regardless of genotype or treatment (p < .0001, repeated measures ANOVA main effect of training; no main effect of genotype/treatment). (B) To evaluate long‐term memory (24 h), probe trials were conducted prior to start of training on day 4 and 24 h after the end of training (day 13). During the probe trials, the platform was removed, and mice were allowed to swim for a total of 60 s. The percentage of time that mice spent searching in the quadrant of the pool where the platform was previously located (target quadrant) was calculated as a measure of spatial memory. During the probe trial conducted on day 4 (first arrow in panel A), WT mice exhibited a selective search strategy, spending significantly more time in the target quadrant (*p < .05, 1‐sample t‐test against chance [25%: dashed line in figure]) whereas the 5XFAD mice exhibited a random search strategy regardless of treatment. During the probe trial carried out on day 13, all mice exhibited a selective search strategy (*p < .05, 1‐sample t‐test against chance [25%: dashed line in figure]). Furthermore, performance in the Morris water maze appeared to be unaffected by se [file CTM2-12-e1046-s004.png]
