# Supplementary material for: Monoclonal antibody‐mediated immunosuppression enables long‐term survival of transplanted human neural stem cells in mouse brain
Source: Clin Transl Med. 2022 Sep 13;12(9):e1046. doi: 10.1002/ctm2.1046 (PMC9471059; doi:10.1002/ctm2.1046)
Supplement: Supplementary file 4 — TABLE S1: Histopathologic toxicity screen on stem cell injected C57BL/6J mice. Histopathologic analysis was performed to screen for toxicity from stem cell treatment (Group A = 3.6 × 10 5 cells, Group B/C = 6.0 × 10 5 cells, Group D = 9.6 × 10 5 cells) and dual mAb immunosuppression at 6 months post‐hNSC transplantation. In examined tissues, no significant findings were noted (‐) except for occasional findings in liver of focal mononuclear infiltration or centrilobular necrosis (†background findings in mice) or portal vein hypoplasia/hepatic arteriolar duplication (‡background finding in C57BL/6J mice). [file CTM2-12-e1046-s002.docx]

| Animal group (designation) | A(0) | A(a) | A(c) | A(f) | B(0) | C(0) | C(a) | C(f) | D(0) | D(a) | D(d) | D(f) |
| --- | --- | --- | --- | --- | --- | --- | --- | --- | --- | --- | --- | --- |
| Brain | - | - | - | - | - | - | - | - | - | - | - | - |
| Heart | - | - | - | - | - | - | - | - | - | - | - | - |
| Liver | - | - | - | - | - | - | - | - | - | - | - | - |
| Infiltration, mononuclear, focal^†^ | - | - | Mild | - | - | - | - | - | - | - | - | - |
| Necrosis, centrilobular^†^ | - | - | - | - | - | Mild | - | - | - | - | - | - |
| Portal vein hypoplasia & hepatic arteriolar duplication^‡^ | - | Moderate | Severe | - | - | - | - | Moderate | - | - | - | - |
| Lungs | - | - | - | - | - | - | - | - | - | - | - | - |
| Spleen | - | - | - | - | - | - | - | - | - | - | - | - |
| Pancreas | - | - | - | - | - | - | - | - | - | - | - | - |

Supplementary Table 1: Histopathologic toxicity screen on stem cell injected C57BL/6J mice.

Histopathologic analysis was performed to screen for toxicity from stem cell treatment (Group A = 3.6x10^5^ cells, Group B/C = 6.0x10^5^ cells, Group D = 9.6x10^5^ cells) and dual mAb immunosuppression at 6 months post hNSC transplantation. In examined tissues, no significant findings were noted (-) except for occasional findings in liver of focal mononuclear infiltration or centrilobular necrosis (^†^background findings in mice) or portal vein hypoplasia/hepatic arteriolar duplication (^‡^background finding in C57BL/6J mice).
